# Discovery of genes coding for carbohydrate-active enzyme by metagenomic analysis of lignocellulosic biomasses

**DOI:** 10.1038/srep42623

**Published:** 2017-02-15

**Authors:** Salvatore Montella, Valeria Ventorino, Vincent Lombard, Bernard Henrissat, Olimpia Pepe, Vincenza Faraco

**Affiliations:** 1 Department of Chemical Sciences, University of Naples “Federico II”, Complesso Universitario Monte S. Angelo, via Cintia, 4 80126 Naples, Italy; 2 Department of Agricultural Sciences, University of Naples “Federico II”, Portici (Napoli), Italy; 3 CNRS UMR 7257, Aix-Marseille University, 13288 Marseille, France; 4 INRA, USC 1408 AFMB, 13288 Marseille, France; 5 Department of Biological Sciences, King Abdulaziz University, Jeddah, Saudi Arabia

## Abstract

In this study, a high-throughput sequencing approach was applied to discover novel biocatalysts for lignocellulose hydrolysis from three dedicated energy crops, *Arundo donax, Eucalyptus camaldulensis* and *Populus nigra*, after natural biodegradation. The microbiomes of the three lignocellulosic biomasses were dominated by bacterial species (approximately 90%) with the highest representation by the *Streptomyces* genus both in the total microbial community composition and in the microbial diversity related to GH families of predicted ORFs. Moreover, the functional clustering of the predicted ORFs showed a prevalence of poorly characterized genes, suggesting these lignocellulosic biomasses are potential sources of as yet unknown genes. 1.2%, 0.6% and 3.4% of the total ORFs detected in *A. donax, E. camaldulensis* and *P. nigra,* respectively, were putative Carbohydrate-Active Enzymes (CAZymes). Interestingly, the glycoside hydrolases abundance in *P. nigra* (1.8%) was higher than that detected in the other biomasses investigated in this study. Moreover, a high percentage of (hemi)cellulases with different activities and accessory enzymes (mannanases, polygalacturonases and feruloyl esterases) was detected, confirming that the three analyzed samples were a reservoir of diversified biocatalysts required for an effective lignocellulose saccharification.

The main renewable resources available to counteract the high greenhouse gas emissions and dependence on feedstock imports associated with fossil sources utilization^1^ are waste materials such as crop and forestry residues, agro-industrial wastes and municipal solid waste^2^ and dedicated energy crops, such as miscanthus, switchgrass, reed canary, giant reed, poplar, willow and eucalyptus^3^,^4^. However, the main drawback of their use is related to the complexity of macromolecular composition that requires an effective disarraying of recalcitrant lignin and a suitable tailor-made enzyme mixture based on (hemi)cellulases and auxiliary enzymes needed to obtain an effective saccharification^5^,^6^. Enzymes involved into the degradation, modification, or creation of glycosidic bonds are referred to as carbohydrate-active enzymes (CAZymes) that are categorized in different classes and families including glycoside hydrolases (GHs), key enzymes for lignocellulosic biomass degradation, glycosyltransferases (GTs), polysaccharide lyases (PLs), carbohydrate esterases (CEs) and carbohydrate-binding modules (CBMs)^7^. The cellulases hydrolyze the *β* (1 → 4) glycosidic bonds and are grouped into three main groups, according to their reaction mechanism: the endoglucanases (EC 3.2.1.4) cut randomly the internal glycosidic bonds in the amorphous cellulose; the exocellulases act from the reducing ends (EC 3.2.1.176) or non-reducing ends (EC 3.2.1.91) of cellulose; the β-glucosidases (EC 3.2.1.21) are involved in the hydrolysis of cellobiose. The hemicellulases include several enzymes–such as endo/exo-xylanases (E.C. 3.2.1.8/37), endo/exo-β-glucanases (EC 3.2.1.6/58), β-mannanases (EC 3.2.1.78), polygalacturonases (EC 3.2.1.15, 67, 82), pectin lyases, pectate lyases (EC 4.2.2.2, 6, 9, 10), pectin methyl esterases (EC 3.1.1.11), arabinofuranosidases (EC 3.2.1.55), feruloyl esterases (EC 3.1.1.73)–acting on specific glyco-units and glycosidic bonds towards different hemicelluloses. Furthermore, auxiliary enzymes acting towards recalcitrant highly crystalline cellulose by a non-hydrolytic mechanism, such as lytic polysaccharides monooxygenases, are needed to enhance the fermentable sugars yield^8^.

Although different combinations of processes for conversion of dedicated energy crops and waste materials into fermentable sugars have been widely studied^9^,^10^,^11^,^12^,^13^,^14^,^15^, the saccharification step is still the main bottleneck in the biorefinery^16^ due to the high costs of the enzyme production and the need for biocatalysts that are efficient and stable at the operative conditions^17^. Therefore, the discovery of novel biocatalysts that could satisfy these criteria is one of the main challenges to overcome this bottleneck. At present, the most advanced researches exploit metagenomes, namely genomic DNAs extracted directly from different environments^18^, bypassing the need for culture under laboratory conditions and avoiding the restrictions related to *in vitro* techniques. Two different methods can be used to screen the metagenomes. The function-driven strategy is performed by a biological activity- screening of expression libraries^18^. The sequence-driven approach is based on the direct sequencing of all genetic material from a target environment and on the homology analysis in comparison with sequences already present in the databases^18^. The increasing number of works focusing on the study of microbiota from guts of wood-eating insects^19^, cow^20^, green-waste compost^21^ shows the relevance of the research for new lignocellulolytic microorganisms and enzymes. At the present, among natural environments, decaying lignocellulosic materials could represent an important reservoir of novel genes encoding enzymes involved in (hemi)cellulose degradation, necessary for the development of eco-compatible and economically favorable industrial processes. In a previous study^22^, new multifunctional degrading bacteria that were potential producers of multiple enzymes that have synergistic actions on cellulose and hemicellulose were isolated and selected from lignocellulosic biomasses using a cultural-dependent approach.

Therefore, in the present work, a sequence-driven metagenomic approach was applied to the three dedicated lignocellulosic energy crops *Arundo donax, Eucalyptus camaldulensis* and *Populus nigra* after natural biodegradation to identify candidate genes coding for enzymes that may be of use in lignocellulose hydrolysis. Moreover, metagenomic DNA sequences were also analysed to assess the complex microbial community structure and taxonomic diversity of the analyzed biomasses and to evaluate the microbial diversity related to GH families of predicted ORFs.

This study provides high-quality results for the identification of sequences coding for enzymes involved in breakdown, biosynthesis or modification of complex carbohydrates such as lignocellulosic biomass.

The data obtained in this work indicate that the investigated feedstock represent a source of biocatalysts potentially suitable for industrial applications to enhance the conversion of lignocellulosic crops into fermentable sugars.

## Results

### Data Statistics

The microbiota of three different lignocellulosic biomasses were analysed by Illumina sequencing of the metagenomic DNAs. A total of 11,208,388,400, 11,274,127,600 and 2,392,000 raw reads for *A. donax, E. camaldulensis* and *P. nigra*, respectively, were obtained. Sequence reads accounting for around 10.0 Gb, for *A. donax* and *E. camaldulensis* samples, and 2 Gb, for *P. nigra*, were selected (Table 1).

The reads were assembled into 95,292, 159,184 and 33,805 contigs (cut-off value 500 bp) for *A. donax, E. camaldulensis* and *P. nigra* biomasses, respectively (Table 1). The N50 and N90 contig lengths ranged from 914 to 1,452 and from 546 to 583 bases, respectively. The longest contig was 49,245, 650,642 and 85,030 bases in *A. donax, E. camaldulensis* and *P. nigra*, respectively (Table 1).

### Microbial community composition of lignocellulosic biomasses

The reads were compared against sequences in the NCBI NR database and the results processed by MEGAN version 4.70.4 to determine the composition of the microbial communities. The three lignocellulosic biomass samples were shown dominated by *Proteobacteria* and *Actinobacteria*. These phyla together accounted for approximately 87.5%, 87.2% and 89.4% of the total biodiversity in *A. donax, E. camaldulensis* and *P. nigra*, respectively (Table 2). In *P. nigra* biomass, *Firmicutes*, and in particular *Bacilli*, were detected at a high incidence (approximately 10%).

A low percentage of reads matched fungal species in *A. donax* and *E. camaldulensis* (2.7% and 5.3%, respectively) (Table 2).

The relative abundances of microbial taxa were examined at the level of genera to determine the dominant taxa within the bacterial communities degrading biomass from the different investigated plant species. The composition of prokaryotic and eukaryotic subpopulations within the biomass were also separately assessed and presented below.

**Figure 1:**
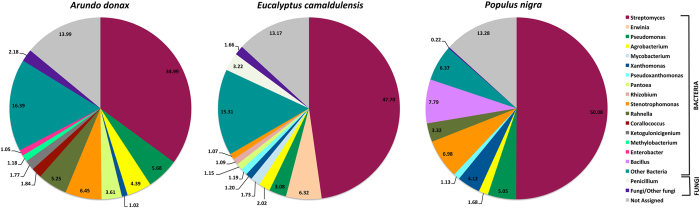
Abundance of bacterial and fungal genera in *A. donax, E. camaldulensis* and *P. nigra* lignocellulosic biomass. Only taxa with an incidence ≥1% in each sample are shown. Other bacteria and other fungi represent the aggregate of other bacterial and fungal genera, respectively; not assigned means that these reads cannot be annotated at the genus level.

In total, sixteen different bacterial genera with an incidence ≥1% were detected in the biomass materials, but only *Streptomyces, Pseudomonas, Agrobacterium, Xanthomonas* and *Stenotrophomonas* were detected in all samples (Fig. 1). In particular, the composition of microbial community in the *P. nigra* biomass was strongly dominated by *Streptomyces* (50.1%), followed by *Bacillus* (7.8%), *Stenotrophomonas* (7%), *Pseudomonas* (5.1%), *Xanthomonas* (4.2%), *Rahnella* (3.3%), *Agrobacterium* (1.7%) and *Pseudoxanthomonas* (1.1%).

As in the *P. nigra* biomass, *Streptomyces* was the taxa that heavily dominated the microbial community in *A. donax* and *E. camaldulensis* (35.0% and 47.7%, respectively), followed by *Pseudomonas, Agrobacterium, Xanthomonas, Pantoea* and *Stenotrophomonas*. The relative abundance of these taxa was very variable showing a percentage ranging approximately from 1% to 6.5%, depending on lignocellulosic plant species (Fig. 1).

In the *E. camaldulensis* biomass, *Erwinia* occurred at a high incidence (6.3%); while, *Corallococcus* (1.8%), *Ketogulonicigenium* (1.8%), *Methylobacterium* (1.2%) and *Enterobacter* (1.1%) genera were recovered only in the *A. donax* biomass (Fig. 1).

The relative abundances of fungal taxa accounted for 2.2%, 4.9% and 0.2% of the total biodiversity in *A. donax, E. camaldulensis* and *P. nigra*, respectively (Fig. 1). In detail, the incidence of all fungal genera identified in *A. donax* and *P. nigra* biomass was <1%; while in *E. camaldulensis* biomass, *Penicillium* strongly dominated the eukaryotic biodiversity showing a relative abundance of 3.2% (Fig. 1).

### eggNOG and KEGG functional profiling of lignocellulosic biomass

With the aim to investigate the functional diversity in the three samples, the predicted amino acid sequences were also aligned to the databases Evolutionary genealogy of Genes non-supervised orthologous groups–eggNOG–and Kyoto Encyclopedia of Genes and Genomes–KEGG–by using BLAST.

**Figure 2:**
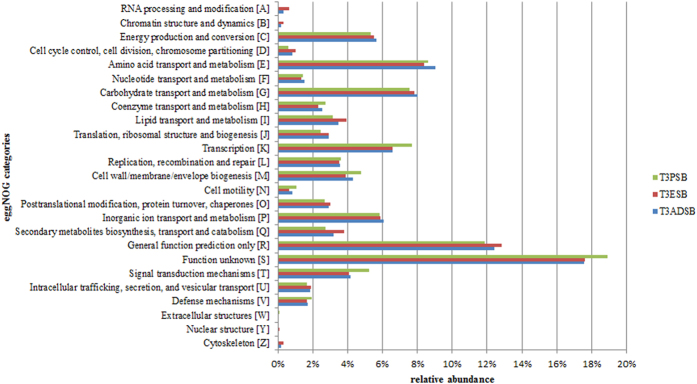
Relative abundance of eggNOG categories related to the predicted ORFs from T3ADSB, T3ESB and T3PSB sample.

As shown in Fig. 2, the data revealed a prevalence of poorly characterized genes belonging to S (function unknown) or R (general function prediction only) eggNOG category. Moreover, for all three samples, a high percentage (~38%) of genes matching to non-supervised orthologous groups were classified involving in metabolism (categories C, E, F, G, H, I, P, Q) with ~8% of genes related to the carbohydrate transport and metabolism.

**Figure 3:**
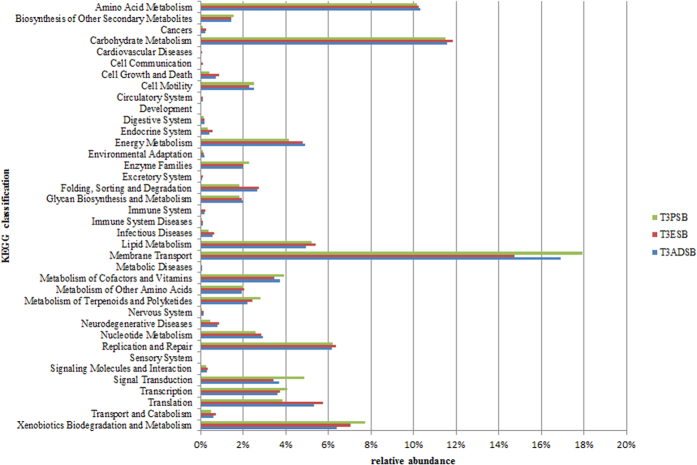
KEGG pathway classification of the predicted ORFs from T3ADSB, T3ESB and T3PSB samples.

As shown in Fig. 3, although the majority of predicted ORFs were related to the membrane transport, this analysis confirmed that many genes matching to KEGG database (~12%) originated from pathways involved in the carbohydrate metabolism.

### Inventory of the detected Carbohydrate-Active Enzymes families and putative plant-polysaccharides-targeting Glycoside Hydrolases

In order to identify putative genes and enzymes involved in breakdown, biosynthesis or modification of carbohydrates, the total predicted ORFs in the three investigated biomass samples were compared to the entries of the Carbohydrate-Active Enzymes (CAZymes) database. A total of 1792, 1279 and 2113 putative CAZymes were identified in the samples T3ADSB (from *A. donax* after 135 days of natural biodegradation in underwood), T3ESB (from *E. camaldulensis* after 135 days of natural biodegradation in underwood) and T3PSB (from *P. nigra* after 135 days of natural biodegradation in underwood) respectively, corresponding to 1.2%, 0.6% and 3.4% of the total ORFs (Table 3). A high relative abundance (25–26%) of predicted CAZymes was reported belonging to glycosyltransferases (GTs) families and involved in forming glycosidic bonds for the biosynthesis of di-, oligo- and polysaccharides. A less amount of Carbohydrate Esterases–CEs–(~5–7%), Polysaccharide Lyases–PLs–(~1–3%) and Auxiliary Activities–AAs- (~2–4%) enzymes were detected in the three samples. Moreover, ORFs coding for putative Carbohydrate-binding modules (CBMs) having binding activity to carbohydrates were 5.2%, 11.6% and 13.5% on total CAZymes for T3ADSB, T3ESB and T3PSB, respectively. Around half of the detected CBMs (2.5%, 6.3% and 7% on total CAZYmes for T3ADSB, T3ESB and T3PSB, respectively) was in conjunction with other non-catalytic CBMs and/or with catalytically-active GHs modules exhibiting a modular structure. In particular, in the metagenome from *A. donax*, most of the multimodular CAZymes contained two modules. Only 4 ORFs encoding putative multimodular proteins contained three modules. In the metagenome from *E. camaldulensis*, multimodular CAZYmes containing CBM32 module were mainly detected. The members belonging to CBM family 32, commonly found in bacterial CAZymes that modify plant cell wall polysaccharides and eukaryotic glycans, were reported to have different substrate specificity^23^. Modular proteins containing CBM32 module were mainly detected even in metagenome from *P. nigra* in multiple copies within the same enzyme or in conjunction with other CBM and/or GH motifs. In this sample, the largest amount of multimodular CAZymes was recognized. In particular, one ORF consisted of seven modules (GH16-CBM4-CBM4-CBM4-CBM4-CBM32-CBM32), one of six modules (CBM54-GH16-CBM4-CBM4-CBM4-CBM4) and one of five modules (CBM35- CBM35- CBM35- CBM35-GH43).

**Figure 4:**
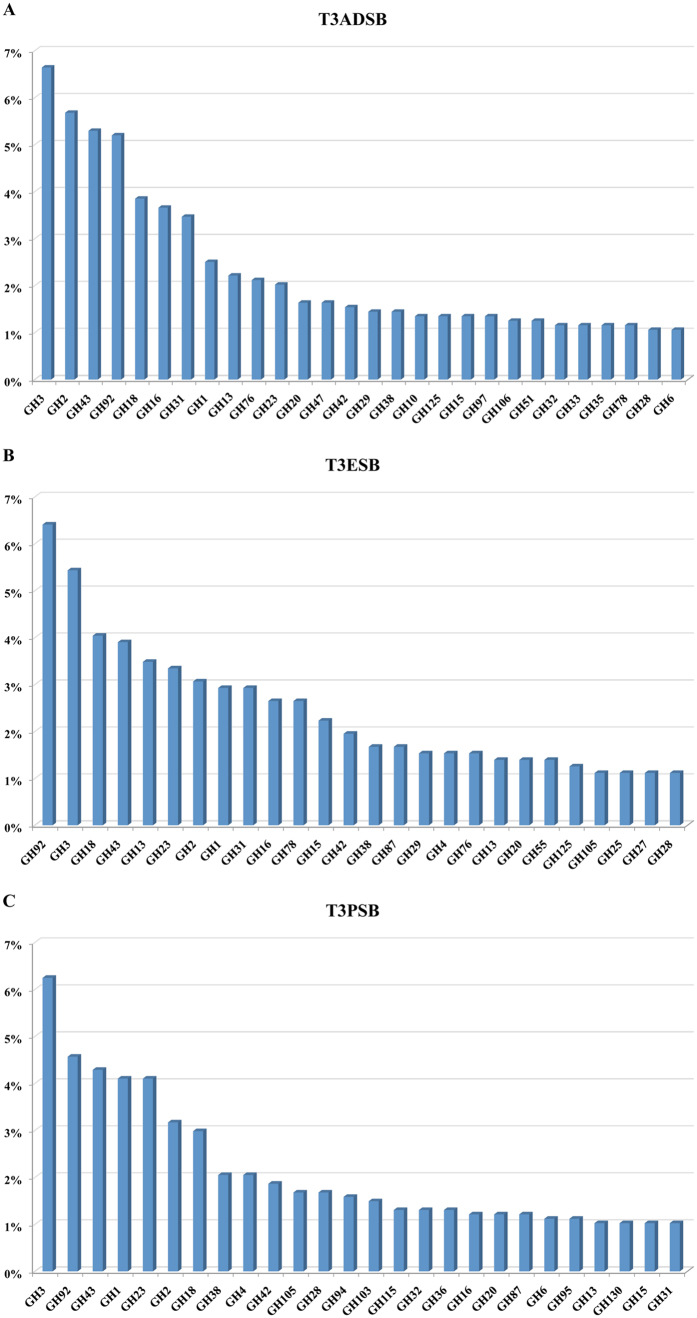
GH family percentage of predicted ORFs from T3ADSB (A), T3ESB (B) and T3PSB (C) samples. The GHs with more than 1% of abundance are reported.

However, most of the detected CAZymes in the three samples were involved in hydrolysis and/or rearrangement of glycosidic bonds. In particular, a number of 1059 in *A. donax* (corresponding to 59.1% on total CAZymes and to 0.7% on total ORFs detected), 750 in *Eucalyptus camaldulensis* (corresponding to 58.6% on total CAZymes and to 0.3% on total ORFs detected) and 1136 in *Populus nigra* (corresponding to 53.8% on total CAZymes and to 1.9% on total ORFs detected) predicted proteins were classified as GHs. The Fig. 4 shows the most frequently occurring putative GHs detected in T3ADSB, T3ESB and T3PSB samples. For each sample, the GHs with abundance ≥1% of the total detected GHs are reported. Table 4 shows the comparison of GH family percentage (abundance >3%) of predicted ORFs from the samples. An abundance of putative GH92–exo-acting α-mannosidases–(5.2%, 6.4% and 4.6% for T3ADSB, T3ESB and T3PSB, respectively, GH3 (6.6%, 5.4% and 6.2% T3ADSB, T3ESB and T3PSB, respectively) and GH43 (5.3% 3.9% and 4.3% T3ADSB, T3ESB and T3PSB, respectively) was noted in all samples. Moreover, in the sample from *Arundo donax* and *Eucalyptus camaldulensis*, a large amount of GH18 (3.9% and 4. % respectively) was detected. This family is reported to include both chitinases and endo-β-N-acetylglucosaminidases but also sub-families of non-hydrolytic proteins. In the metagenome from *Eucalyptus camaldulensis*, CAZymes belonging to family GH13 were relatively abundant. The GH13 enzymes act on a wide range of different substrates and have been subdivided into almost 40 subfamilies, most of which are monofunctional^24^. In particular, in all three samples, only GH13 belonging to subfamily 11 (reported having debranching activity on glycogen, amylopectin and their β-limit dextrins) and subfamily 30 (involved in the hydrolysis of terminal α-D-glucose residues with release of monomers) were detected. Moreover, the sample from *Populus nigra* showed a high abundance of GHs belonging to GH23 family (4.1%). All the enzymes belonging to GH23 family were reported to have activity on peptidoglycan and, in particular, the lysozymes to have activity even on chitin and chitooligosaccharides.

**Figure 5:**
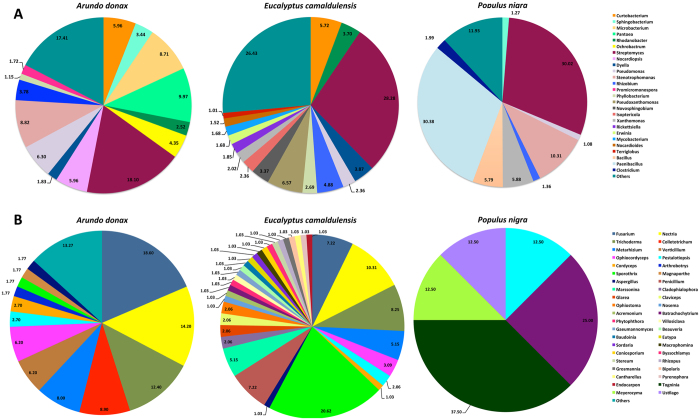
Percentage composition of bacterial (**A**) and fungal (**B**) genera related to GH families of predicted ORFs in *A. donax, E. camaldulensis* and *P. nigra biomass*. Only taxa with an incidence ≥1% in each sample are shown. Others represent the aggregate of other bacterial (**A**) and fungal (**B**) genera.

The microbial diversity of the ORFs predicted to encode GHs from the three lignocellulosic biomasses was also investigated to identify the bacterial and fungal genera encoding enzymes involved in the carbohydrate metabolism. The microbial biodiversity related to GHs was very high and twenty-six bacterial and forty-two fungal genera were recovered with an incidence ≥1% in at least one sample (Fig. 5). *Streptomyces* was a dominant genus in all samples accounting for 18.1%, 28.3% and 30.0% in *A. donax, E. camaldulensis* and *P. nigra*, respectively, of the microbial genera related to GH (Fig. 5A).

Unlike the other biomasses in which *Streptomyces* was the dominant taxon, in *P. nigra* the most abundant GHs were related to *Paenibacillus* (30.38%) (Fig. 5A). *Pseudomonas* and *Rhizobium* were the other genera recovered in all lignocellulosic biomasses in relationship to the GHs (Fig. 5A) showing an abundance ranging from 1.1% to 6.3% depending on plant species.

The abundance of the other taxa related to GHs is strictly correlated to substrate source. A high percentage of bacteria belonging to *Stenotrophomonas* genus encoded GHs in *A. donax* (8.8%) and *P. nigra* (10.3%) biomass (Fig. 5A). Also most GHs in *A. donax* biomass was encoded by genera belonging to the class of *Actinobacteria* and in particular, *Curtobacterium* (6.0%), *Microbacterium* (8.7%), *Nocardiopsis* (6.0%) and *Promicromonospora* (1.2%) (Fig. 5A). In contrast, members belonging to α-*Proteobcteria (Novosphingobium* and *Isoptericola*) and γ-*Proteobacteria (Pseudoxanthomonas, Xanthomonas, Dyella* and *Rhodanobacter*) classes characterized *E. camaldulensis* biomass; while γ-*Proteobacteria (Stenotrophomonas* and *Xanthomonas*) together to *Bacillus* (5.8%) were the other taxa recovered in the *P. nigra* biomass (Fig. 5A).

In this study, the GHs also originated from a wide range of fungal taxa. Among the forty-two genera occurring with an abundance ≥1% in at least one sample, only *Pestalotiopsis* was recovered in all lignocellulosic biomasses (with an incidence of 2.7%, 2.0% and 12.50 in *A. donax, E. camaldulensis* and *P. nigra*, respectively) (Fig. 5B).

Overall, the highest fungal biodiversity related to GHs was found in *E. camaldulensis* (35 genera) followed by *A. donax* (13 genera) and *P. nigra* (5 genera). Although the highest biodiversity was found in *E. camaldulensis*, all fungal genera occurred at low percentage with the exception of *Nectria* (10.3%) and *Sporothrix* (20.6%) (Fig. 5B). By contrast, in *A. donax* biomass, most of the GHs were related to *Fusarium* (18.6%), *Nectria* (14.2%) and *Trichoderma* (12.4%), while the abundance of the other taxa range approximately from 8% to 1% (Fig. 5B).

Finally, the lowest fungal diversity was found in *P. nigra* biomass. The most abundant taxa recovered in this plant sample was *Togninia* (37.5%) followed by *Batrachochytrium* (25.5%) and *Pestalotiopsis, Meyerozyma* and *Ustilago* (12.5%) (Fig. 5B). However, although this result seemed suggest that the fungal taxa were abundant, overall very few GHs were related to them because only the 0.2% of total biodiversity was determined by fungi in this sample (Fig. 1).

### KEGG pathway classification related to Glycoside Hydrolases

**Figure 6:**
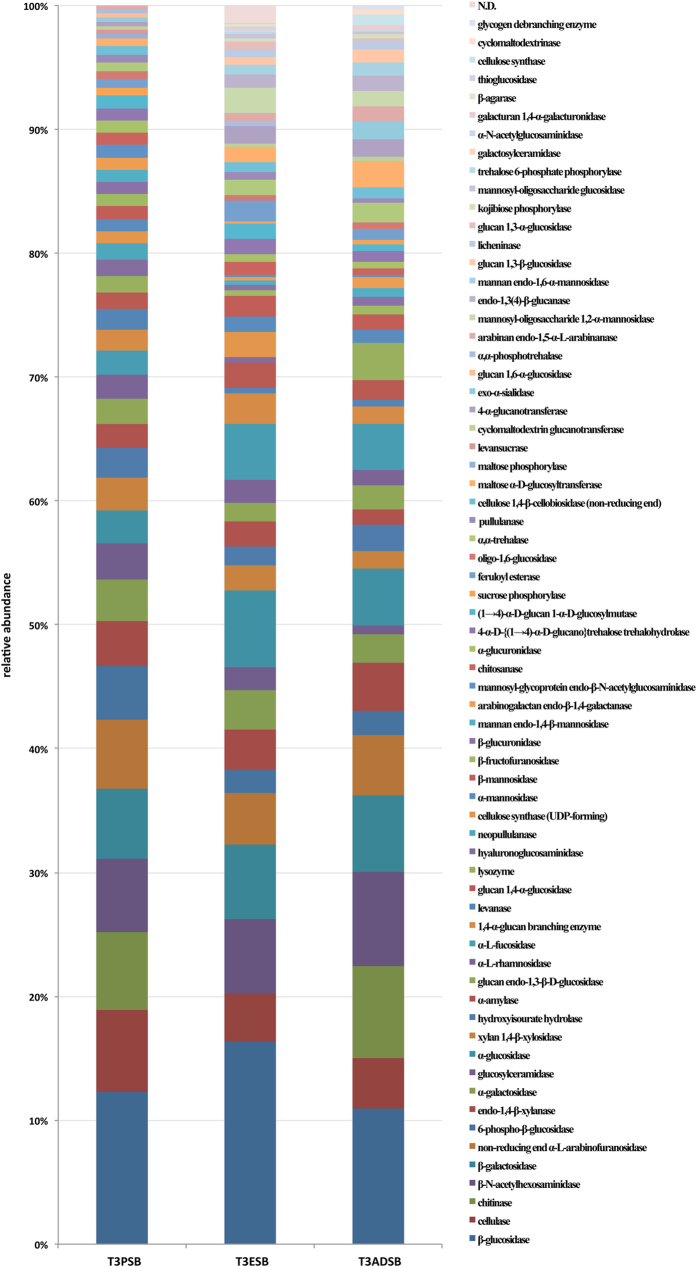
KEGG pathway classification for the putative genes coding for Enzyme Commission (EC) number activities related to the hydrolysis of glycosidic bonds from T3ADSB, T3ESB and T3PSB samples.

An in-depth KEGG pathway mapping was carried out for the putative genes coding for plant polysaccharides-degrading enzymes in order to obtain a specific, unique activity for each detected GH. As shown in Fig. 6, a high percentage of different cellulases were detected. In particular, β-glucosidases (EC 3.2.1.21, hydrolyzing cellobiose and other cellodextrins) and endo-1,4-β-glucanases (EC 3.2.1.4, performing the random internal hydrolysis of amorphous cellulose) were the most abundant putative enzymes involved in the hydrolysis of glycosidic bonds. In the samples from *Arundo donax* and *Populus nigra,* an abundance of chitinases (EC 3.2.1.14) was also detected (7.41% and 6.29% respectively). It is noteworthy that in all three samples putative genes coding for hemicellulases and accessory enzymes with a broad spectrum of activities were recognized. In particular, a high percentage of proteins involved in the degradation of (glucurono)(arabino)xylan–such as endoxylanases (E.C. 3.2.1.8) and β-xylosidases–and in the removal of arabinose–α-L-arabinofuranosidases (E.C. 3.2.1.55)–or galactose–α-galactosidases (E.C. 3.2.1.22)–substituents in hemicelluloses were detected. Moreover, several additional putative enzymes related to the hemicelluloses degradation–such as mannanases (EC 3.2.1.78), polygalacturonases (EC 3.2.1. 67) and feruloyl esterases (EC 3.1.1.73) were recognized in a lower percentage.

## Discussion

In the last decades, the increasing interest in the use of renewable sources for green energy and chemicals has strongly stimulated search for new biocatalysts from different ecosystems for lignocellulose conversion. Therefore, in this work, microbial and enzymatic diversities potentially relevant to the degradation of plant biomass into fermentable sugars were explored through metagenomic approach in three dedicated lignocellulosic energy crops, *Arundo donax, Eucalyptus camaldulensis* and *Populus nigra*, after natural biodegradation^22^. Metagenomic DNA sequences were analysed to assess the total biodiversity, identify candidate genes coding for enzymes putatively involved in carbohydrates metabolism and that may be of use in lignocellulosic degradation, and evaluate microbial diversity related to GH families of predicted ORFs.

The microbial diversity results from this study were performed on the same samples previously characterised using 16S phylotyping in our earlier study^22^ with samples T3ADSB, T3ESB and T3PSB corresponding to samples At3UW, Et3UW and Pt3UW in that publication. Some taxa differed sharply in composition, e.g. Actinobacterial content of 40.1% *vs* 8.6% when T3ADSB and At3UW were compared. The substantial differences could be due to the different molecular methods adopted by Ventorino *et al*.^22^ for sequencing in comparison to those ones used in this study (amplicon sequencing of the 16S rRNA gene *vs* shotgun metagenomic sequencing) as well as to the different methods used for microbial DNA extraction. In fact, in the present work eDNA was extracted directly from lignocellulosic biomass samples, whereas Ventorino *et al*.^22^ extracted DNA from pellets obtained from microbial cells desorbed from lignocellulosic materials. This approach could determine an underrepresentation of filamentous bacteria, and in general of relative abundance of *Actinobacteria*, in amplicon data reported in the previous work. Discrepancies between different approaches to quantifying the taxonomic composition of microbiomes are a known phenomenon. According to Morgan *et al*.^25^ the relative abundances of microbial taxa inferred from metagenomic sequences significantly varied depending on the DNA extraction and sequencing protocols utilized. Recently, Duncan *et al*.^26^ revealed that shotgun metagenomics detected a much higher abundance of *Actinobacteria* than amplicon sequencing.

Nevertheless, *Actinobacteria* were significant components of biomass in both studies. The prevalence of the actinobacterial genus *Streptomyces* could be due to the ability to synthetize enzymes, such as cellulases^27^,^28^, which efficiently degrade lignocellulosic materials under a wide range of environmental conditions^29^. *Actinobacteria*, and in particular, *Streptomyces* spp. were found to be major plant biomass degrading microbes in peat swamp forests and also ubiquituously present during the composting of chestnut green waste^30^,^31^.

Bacterial species belonging to *Proteobacteria* phylum, such as *Pseudomonas* spp. and *Stenotrophomonas* spp., were also retrieved in all lignocellulosic samples. Bacteria belonging to these genera are known to be able to produce a wide range of enzymes for efficient degradation of carboxymethylcellulose, (hemi)cellulose and lignin^32^,^33^. These results are in according with previous study in which culture-independent approach based on 16S rRNA gene sequence demonstrated that *Proteobacteria* was the taxa that heavily dominated the microbial community in different lignocellulosic biomass piles, remaining high during all degradation processes in natural conditions^22^. Moreover, *Actinobacteria* and *Proteobacteria* have been identified as the predominant bacterial phyla during composting of lignocellulosic waste exhibiting the enzymatic activities required for the degradation of this recalcitrant polymeric material^34^.

The occurrence of other bacterial taxa with a different abundance depending on plant species was also demonstrated in the investigated lignocellulosic biomasses. Interestingly, *Bacillus* genus covered approximately 8.0% of the total microbial biodiversity in *P. nigra*. Members belonging to *Bacillus* spp. isolated from different environments exhibit cellulolytic and/or hemicellulolytic activities to potentially breakdown the components of lignocellulosic material^35^,^36^,^37^,^38^. Moreover, different microbial strains belonging to *Enterobacteriaceae* family such as *Pantoea, Rahnella* and *Erwinia*, are frequently recovered in the gut of insects producing digestive enzymes implicated in the hydrolysis of cellulose^39^,^40^.

Moreover, a low abundance of eukaryotic populations was observed in all the lignocellulosic biomass samples. This result could be due to the fact that fungi have tough chitin walls that are difficult to breach. In fact, since fungal community patterns could be strongly dependent on the extraction method used^41^, their representation in this work could be depressed. However, among the fungal taxa retrieved, only *Penicillium* showed an incidence >1% in *E. camaldulensis*. Cellulolytic activity of this genus is well documented and there are several reports on β-glucosidase, cellulases and xylanases production from different *Penicillium* species^42^,^43^,^44^. Moreover, Ryckeboer *et al*.^45^ reported also the ability of *Penicillium* spp. to degrade lignin and starch making it a good candidate in the producing of industrial cellulases^46^.

Analysing the biodiversity related to GH families of predicted ORFs, a highly complex microbial community was found. With regard to bacterial biodiversity, *Streptomyces, Pseudomonas* and *Rhizobium* were found in all lignocellulosic biomass samples. In agreement with the results obtained analysing the total biodiversity, *Streptomyces* was the dominant taxon, confirming the ability of the members belonging to this genus to encode enzymes involved in cellulose and hemicellulose degradation. In fact, *Streptomyces* spp. is reported to produce different GHs that are well characterized^47^,^48^. In addition, the production of cellulolytic enzymes in *Rhizobium* spp. is related to their ability to nodulate leguminous plants. In fact, *Rhizobium* is a plant growth promoting rhizobacterium living as free-living saprophytes in the soil but also able to fix nitrogen establishing a symbiotic associations with a host plant^49^. The production of enzymes, such as cellulases, is fundamental to degrade plant cell wall polymers and penetrate in the host root^50^. García-Fraile *et al*.^51^ reported the ability to actively hydrolyse CM-cellulose of two bacterial strains isolated from decaying wood of *Populus alba* and classified as *Rhizobium cellulosilyticum*.

The prokaryotic biodiversity related to GHs was also dominated by *Paenibacillus* genus in the *P. nigra* biomass. Eida *et al*.^52^ reported the ability of different *Paenibacillus* isolates to efficiently contribute to cellulolytic and hemicellulolytic processes during composting of sawdust. Other taxa recovered in the *P. nigra* biomass that are known as plant biomass-degrading microbes were *Stenotrophomonas* and *Xanthomonas (Proteobacteria*) and *Bacillus (Firmicutes*). De Angelis *et al*.^17^ reported that the members of *Proteobacteria* as well as *Firmicutes* strongly dominated switchgrass-adapted communities comprising approximately 80% of the microbial richness.

Differently, in *A. donax* biomass the most of GHs was encoded by genera belonging to the class of *Actinobacteria.* These taxa are related to well characterized potent plant polysaccharide-degrading bacteria and play an important role in degradation of numerous polymers such as chitin, cellulose, lignin and polyphenol^53^.

With regard to fungal biodiversity related to GHs, diverse genera were found, and among these only *Pestalotiopsis* was recovered in all lignocellulosic biomasses. This result is in agreement with Cahyani *et al*.^54^ that reported the ubiquitous presence of *Pestalotiopsis* spp. during the composting process of rice straw. In fact, this endophytic fungus is able to secrete xylanases and cellulases also in salt stress conditions^55^ as well as produce a considerable amount of ligninolytic enzymes such as laccase^56^.

However, *Sporothrix, Fusarium, Nectria* and *Trichoderma* dominated the eukaryotic biodiversity related to GHs in *A. donax* and *E. camaldulensis* biomasses. These Ascomycota are known for their ability to produce cellulolytic enzymes^57^,^58^ and comprise many species involved in the degradation of recalcitrant substances such as cellulose, hemicellulose, pectin, and lignin^59^. Jurado *et al*.^60^ reported that fungi belonged to Ascomycota group were ubiquitous throughout the whole lignocellulose-based composting process.

The functional clustering of the predicted ORFs to eggNOG and KEGG databases showed high similarity among the three analyzed samples.

The prevalence of poorly characterized genes obtained by matching to eggNOG categories suggested the three detected biomasses as potential sources of not yet known genes. Moreover, the analysis of functional classification distribution among these three metagenomes, based on both the eggNOG and KEGG database, suggests that a large number of predicted genes were putatively associated with formation, breakdown and interconversion of polysaccharides. In particular, the relative abundance of genes linked to carbohydrates metabolism pathway was higher than or similar to that detected in metagenomes from samples with well-known lignocellulose-degrading ability, such as invasive snail crop microbiome^61^ and lower termite *Coptotermes gestroi* gut^62^. This result confirmed the high potentiality of the three analyzed metagenomes to express genes involved in lignocellulosic biomasses biotransformation.

Moreover, the inventory of the Carbohydrate-Active Enzymes families detected in the three samples interestingly revealed ORFs codifying for putative lytic polysaccharide monooxygenases (LPMOs). Nowadays, the interest is moving towards the LPMOs belonging to AA9 (formerly reported as GH61), AA11 or AA10 (formerly reported as CMB33) families, due to their ability to depolymerize the recalcitrant insoluble polysaccharides from highly crystalline cellulose, increasing the efficiency of lignocellulose saccharification^8^. Only a few of LPMOs have been discovered by metagenomic approach^18^. In metagenomes analyzed in this study, 3, 5 and 9 ORFs (for T3ADSB, T3ESB and T3PSB respectively) were assigned to family AA10, whereas only in the sequenced eDNA from *A. donax* 11 and 2 ORFs encoding putative enzymes belonging respectively to families AA9 and to AA11 were detected.

However, most of CAZymes detected in the three samples were related to putative plant-polysaccharides-targeting GHs. Based on the results obtained by Li *et al*.^63^ analyzing 46 finished metagenomic studies collected in Genomes OnLine Database (GOLD) by comparison against the CAZy sequences for homologues of glycosyl hydrolases using an e-value <10^−40^ as a cut-off threshold, the percentages of detected GHs in our study were higher than those present in metagenomic samples from soil, sludge and marine or lake environments. Furthermore, the diversity of GH family enzymes detected in the three samples was greater than that observed in insect or mammalian fecal and gut samples with high lignocellulose-degrading potentiality^64^, in line with the detected high phylogenetic diversity.

The putative genes encoding proteins involved in the degradation of plant polysaccharides were detected in the three samples. Moreover, accepted that the obtained data are sensitive to the bioinformatics workflow used in the different studies, a comparison between the GHs detected in our samples and in metagenomes well known as reservoirs of genes involved in lignocellulose-degradation was attempted (Table 5), based on the classification provided by Allgaier *et al*.^65^. The detection and assignment of glycoside hydrolases in our metagenomes and bovine rumen metagenome^66^ were performed by BLAST-based procedures against the CAZy database, whilst the searches for glycoside hydrolases in metagenomes from six years old elephant feces^64^, yak^67^ and cow rumen^66^, snail crop^61^, macropod gut^68^ and termite hindgut^19^ were performed by using HMMER hmmsearch with Pfam. The putative ORFs encoding enzymes related to the oligosaccharides degradation represented the majority of the total plant-polysaccharides-targeting GHs and their abundance (~26% for T3ADSB, ~22% for T3ESB and ~24% for T3PSB) was comparable to that detected in samples from cow rumen^20^ and termite hindgut^19^. Most belonged to GH1, GH2 and GH3 families including β-glucosidases, β-galactosidases, β-mannosidase, β-glucuronidase, β-xylosidase and other enzymes involved in the breakdown of a large variety of β-linked disaccharides. Due to the high diversity of protein structural arrangements, a robust phylogenetic classification of these families is currently not available. In addition, enzymes belonging to GH43 family were highly represented (mainly in T3ADSB and T3PSB). This family includes β-xylosidases and α-L-arabinofuranosidases and several bifunctional enzymes; moreover, due to a remarkable expansion in GH43 family resulting from novel studies about plant cell wall degrading organisms, members of this family may have a more extensive range of specificities^69^.

In the sample T3ADSB, the abundance of endocellulases was double than T3ESB and T3PSB and comparable to that detected in the six-years-old elephant feces by Ilmberger *et al*.^64^ and in yak rumen by Dai *et al*.^67^. The GH5 and GH6 were the most represented families. While only endoglucanase and cellobiohydrolase activities have been reported for the members of GH6 family, the enzymes belonging to Glycoside Hydrolases family 5 have a variety of specificities: this is one of the largest of all CAZy glycoside hydrolase families comprising not only cellulases, such as endo- and exo-glucanases and β-glucosidases, but even hemicellulases, such as endo- and exo-mannanases and β-mannosidase. Interestingly, in T3ADSB an amount of enzymes belonging to GH7 family (that includes mainly enzymes from fungi) was detected, although in this sample only a small amount of fungi was identified. The cellobiohydrolases belonging to GH7 family are the most active exoglucanases known^70^.

The abundance of hemicellulases detected in the three investigated samples was comparable with the percentage occurred in bovine rumen^66^ and macropod gut^68^. In T3ADSB, more that 1% of CAZymes belonged to GH10 family. These enzymes have received much attention for their use in degradation of lignocellulosic biomass for biochemicals production, due to their involvement in breaking down of xylan, the major component of the hemicellulose. Moreover, in the three samples a percentage of 1–2% of enzymes belonging to Glycoside Hydrolases family 28 was identified. These CAZymes are involved in the degradation of pectin, a structural constituent of the plant cell wall.

About 1% of the debranching enzymes detected in the three samples belonged to family GH51: this percentage was higher than that detected in yak and cow rumen^20^,^67^ and snail crop^61^. Moreover, the samples T3ESB and T3PSB revealed an abundance of family GH67 members. The enzymes belonging to these two families (α-L-arabinofuranosidases and α-glucuronidases respectively) are required for the optimal breakdown of glucoronoarabinoxylans (GAXs), one of the major component of hemicellulose, composed by β(1–4)-D-xylose linked polymers branched with arabinose and glucuronic acid. Interestingly, in the samples from *Eucalyptus camaldulensis* and *Populus nigra* 2.5% and 0.6% respectively of total GHs belonged to GH78 α-L-rhamnosidases. These enzymes catalyze the hydrolysis of α-L-rhamnosyl-linkages in L-rhamnosides present in polysaccharides such as rhamnogalacturonan.

Furthermore, the in-depth KEGG pathway mapping of the genes encoding enzymes involved in the polysaccharides hydrolysis confirmed that all three analyzed samples were a valuable source of a full set of diversified (hemi)cellulases and accessory enzymes required for an effective pretreated lignocellulosic biomass hydrolysis^71^,^72^.

## Methods

### Lignocellulosic biomasses and DNA extraction

Chipped wood from *A. donax, E. camaldulensis* and *P. nigra* was used to form piles of approximately 30 kg that were submitted to biodegradation under natural conditions as previously reported^22^. Briefly, the biomass piles were placed without any coverage under oak trees in the woodland at the Department of Agriculture (Naples, Italy). After 135 days of natural biodagradation, samples of 0.5 kg were collected from the external part (right and left side of the pile) and the internal central part of the biomass, milled and stored at −20 °C until use.

3 g of each milled biomass were used to isolate the total environmental DNA (eDNA), including genetic material from microorganisms adherent to the plant biomass. The eDNA extraction was performed by using the PowerSoil^®^ DNA Isolation Kit (MO BIO Laboratories, INC. CARLSBAD, CA) according to the manufacturer’s instructions. NanoDrop and Qubit Fluorometer tests were performed to verify the level of purity of recovered eDNA. About 25 μg of each eDNA samples were sent to BGI Tech Solutions Co., Ltd. (Hongkong, China) for further analyses.

### Metagenome shotgun sequencing and assembly

Three qualified 270 bp short-insert libraries were constructed from the eDNA samples. The genetic material was firstly sheared into smaller fragments by nebulization. Then the overhangs resulting from fragmentation were converted into blunt ends by using T4 DNA polymerase, Klenow Fragment and T4 Polynucleotide Kinase. An “A” base was added to the 3′ phosphorylated blunt ends of the DNA fragments and the adapters were ligated. Undersized fragments were removed with Agencourt AMPure XP Beads (Beckman Coulter Inc, Brea, CA, USA). The libraries were then subjected to 151 paired-end sequencing on Illumina HiSeq2000 platform by using TruSeq SBS Kit v3-HS (Illumina, San Diego, CA, USA) following standard pipelines. The generated raw data were trimmed: leading or trailing low quality (below quality 3) or 3 N bases were cut off and reads contaminated by adapter (15 bases overlapped by reads and adapter) or with low quality (20) bases (40% as default, parameter setting at 36 bp) were removed. The data were filtered by using readfq.v5 (unpublished software, BGI).

The obtained Clean Data were used to perform the metagenome sequences. Before assembly, k-mer analysis (K-mer length 15) was done to evaluate the sequencing depth for each sample. SOAPdenovo (Version 1.06)^73^ was used to assemble filtered data in contigs and scaffolds and assembly results were optimized by in-house scripts (key parameters: -r 2; -l 35; -M 4; -p 1) using the SOAP-aligner tool.

### Metagenome analyses

To evaluate the microbial composition, the assembled contigs were matched against the bacteria, fungi and archaea sequences extracted from NCBI NR database (release-20130408) by BLASTx with 1 × 10^−8^ and ≥90% identity cut-off. Each contig was subsequently taxonomically assigned by MEGAN version 4.70.4^74^, based on lowest common ancestor (LCA). The taxonomic abundance was determined by read count of each taxon, after mapping to the assembled contigs using SOAPaligner version 2.21^75^ with default parameters. Assembled contigs are used to predict genes by using MetaGeneMark Software^76^ (version 2.10, default parameters) based on assembly results.

Functional annotations of predicted amino acid sequences were performed by BGI Tech Solutions Co., Ltd. (Hongkong, China) by using BLASTP (version 2.2.23). In particular, the metabolism pathway assignment of the predicted protein was performed using the Enzyme Commission (EC) number in the Kyoto Encyclopedia of Genes and Genomes (KEGG)–version 59–databases^77^ and the annotation of each contig with functional categories was carried out by matching against Evolutionary genealogy of genes: Non-supervised Orthologous Groups (eggNOG)–version 3.0^78^. Both comparison were performed by using BLAST^79^ with e-value threshold of 1e-5 and a 40% minimum percentage of identity to assign the subject sequence to a specific function family. Moreover, in order to explore in depth the ability of the microbial biodiversity detected in the samples to degrade lignocellulose, the putative encoded protein sequences were first compared to the full length sequences of the CAZy database using BLAST^75^ and query sequences that produced a e-value >10^−6^ were discarded. Query sequences that produced an e-value <10^−6^ and aligned over their entire length with a protein in the database with >50% identity were automatically assigned to the same family as the subject sequence. The remaining query sequences were subjected to manual curation which involved BLAST searches against a library built with partial sequences corresponding to individual GH, PL, CE and CBM modules and examination of the conservation of specific family patterns and features such as catalytic residues (where known).

## Additional Information

**Accession codes:** The data is available in the Sequence Read Archive database of the National Center of Biotechnology Information (SRP090993).

**How to cite this article**: Montella, S. *et al*. Discovery of genes coding for carbohydrate-active enzyme by metagenomic analysis of lignocellulosic biomasses. *Sci. Rep.* **7**, 42623; doi: 10.1038/srep42623 (2017).

**Publisher's note:** Springer Nature remains neutral with regard to jurisdictional claims in published maps and institutional affiliations.

## Figures and Tables

**Table 1:** Quality and statistical summary of sequencing and assembling.

Parameter	Biomass sample		
	*A. donax*	*E. camaldulensis*	*P. nigra*
Total reads (bp)	11,208,388,400	11,274,127,600	2,392,000
High quality reads (bp)	10,010,000,000 (89%)	10,010,000,000 (88%)	2,100,000 (88%)
Number of contigs	95,292	159,184	33,805
Length of contigs (bp)	111,530,551	143,424,210	40,937,098
N50 contig length (bp)	1,326	914	1,452
N90 contig length (bp)	574	546	583
Largest contig (bp)	49,245	650,642	85,030
Shortest contig (bp)	500	500	500
Mapping			
PE	6,497,524	7,683,451	2,852,867
SE	1,790,597	2,097,376	1,253,515
Total (%)	14.77	17.45	33.14

**Table 2:** Relative abundance of dominant taxa at the phylum and class rank mapping the high quality reads to the NT database (NCBI).

	Arundo donax		Eucalyptus camaldulensis		Populus nigra	
	Phylum (%)	Class (%)	Phylum (%)	Class (%)	Phylum (%)	Class (%)
Bacteria	Proteobacteria (47.4%)	γ-Proteobacteria (27.1%)	Proteobacteria (29.6%)	γ-Proteobacteria (17.4%)	Proteobacteria (29.6%)	γ-Proteobacteria (25.2%)
		α-Proteobacteria (13.5%)		α-Proteobacteria (9.0%)		α-Proteobacteria (3.2%)
		β-Proteobacteria (4.0%)		β-Proteobacteria (2.9%)		β-Proteobacteria (1.0%)
		δ-Proteobacteria (2.4%)				
	Actinobacteria (40.1%)	Actinobacteria (40.1%)	Actinobacteria (57.6%)	Actinobacteria (57.6%)	Actinobacteria (59.8%)	Actinobacteria (59.8%)
	Bacteroidetes (1.0%)				Firmicutes (10.4%)	Bacilli (10.3%)
Fungi	Ascomycota (2.7%)	Sordariomycetes (1.6%)	Ascomycota (5.3%)	Eurotiomycetes (3.4%)		
				Sordariomycetes (1.1%)		

Only taxa with an incidence ≥1% in each sample are shown.

**Table 3:** CAZYmes classification of predicted ORFs from T3ADSB, T3ESB and T3PSB sample.

CAZymes classification	T3ADSB		T3ESB		T3PSB	
	# ORFs	%	# ORFs	%	# ORFs	%
Auxiliary Activities enzymes (AAs)	76	4.2%	23	1.8%	45	2.1%
Carbohydrate-binding modules (CBMs)	94	5.2%	148	11.6%	285	13.5%
Carbohydrate Esterases (CEs)	110	6.1%	68	5.3%	159	7.5%
Glycoside Hydrolases (GHs)	1059	59.1%	750	58.6%	1136	53.8%
Glycosiltransferases (GTs)	460	25.7%	320	25.0%	555	26.3%
Polysaccharide Lyases (PLs)	24	1.3%	37	2.9%	48	2.3%
Total CAZYmes*	1792*		1279*		2113*	

^*^The total numbers of CAZYmes is less than the sum (AAs + CBMs + CEs + GHs + GTs + PLs) due to the fact that some multimodular predicted proteins were detected.

**Table 4:** Comparison of GH family percentage of predicted ORFs from T3ADSB, T3ESB and T3PSB sample.

CAZy Family	Main Known activities	T3ADSB	T3ESB	T3PSB
GH1	β-glucosidases, β-galactosidases, 6-phospho-β-glucosidase and 6-phospho-β-galactosidase, β-mannosidase, β-D-fucosidase and β-glucuronidase	2.50%	2.92%	4.10%
GH2	β-galactosidases, β-glucuronidases, β-mannosidases, exo-β-glucosaminidases	5.68%	3.06%	3.17%
GH3	exo-acting β-D-glucosidases, α-L-arabinofuranosidases, β-D-xylopyranosidases and N-acetyl-β-D-glucosaminidases	6.64%	5.43%	6.24%
GH16	Xyloglucosyltransferase, keratan-sulfate endo-1,4-β-galactosidase, endo-1,3-β-glucanase, endo-1,3(4)-β-glucanase, licheninase, β-agarase, κ-carrageenase, xyloglucanase, endo-β-1,3-galactanase, β-porphyranase, hyaluronidase, endo-β-1,4-galactosidase, chitin β-1,6-glucanosyltransferase, endo-β-1,4-galactosidase	3.66%	2.65%	1.21%
GH18	chitinases and endo-β-N-acetylglucosaminidases	3.85%	4.04%	2.98%
GH23	muramidase, peptidoglycan N-acetylmuramoylhydrolase, 1,4-β-N-acetylmuramidase and N-acetylmuramoylhydrolase	2.02%	3.34%	4.10%
GH43	α-L-arabinofuranosidases, endo-α-L-arabinanases (or endo-processive arabinanases) and β-D-xylosidases	5.29%	3.90%	4.29%
GH92	exo-acting α-mannosidases	5.20%	6.41%	4.57%

The GHs with an incidence >1% in each sample are shown are reported.

**Table 5:** Comparison of plant polysaccharides hydrolyzing enzymes in our samples T3ADSB, T3ESB and T3PSB and in samples with the highest lignocellulose-degrading potentiality.

CAZy family	Main known activity	Pfam domain	Metagenomic samples																			
			T3ADSB^b^		T3ESB^b^		T3PSB^b^		Six years old elephant feces (Ilmberger *et al*., 2014)^a^		Yak rumen (Dai *et al*., 2012)^a^		Snail crop (Cardoso *et al*., 2012)^a^		Cow rumen (Hess *et al*., 2011)^a^		Bovine rumen(Brulc *et al*., 2009)^b^		Macropod gut (Pope *et al*., 2010)^a^		Termite hindgut (Warnecke *et al*., 2007)^a^	
Endo-Celluases																						
GH5	cellulase	PF00150	40	3.8%	15	2.0%	23	2.0%	517	4.7%	1302	3.5%	36	1.4%	1451	5.2%	7	0.7%	10	1.8%	56	8.0%
GH6	endoglucanase	PF01341	11	1.0%	5	0.7%	12	1.1%	0	0.0%	0	0.0%	4	0.2%	0	0.0%	0	0.0%	0	0.0%	0	0.0%
GH7	endoglucanase	PF00840	11	1.0%	1	0.1%	0	0.0%	0	0.0%	0	0.0%	0	0.0%	1	0.0%	0	0.0%	0	0.0%	0	0.0%
GH9	endoglucanase	PF00759	2	0.2%	3	0.4%	5	0.4%	119	1.1%	767	2.0%	15	0.6%	795	2.9%	6	0.6%	0	0.0%	9	1.3%
GH44	endoglucanase	NA	0	0.0%	0	0.0%	0	0.0%	7	0.1%	0	0.0%	0	0.0%	0	0.0%	0	0.0%	0	0.0%	6	0.9%
GH45	endoglucanase	PF02015	1	0.1%	0	0.0%	0	0.0%	7	0.1%	13	0.0%	0	0.0%	115	0.4%	0	0.0%	0	0.0%	4	0.6%
GH48	endo-processive cellulase	PF02011	1	0.1%	1	0.1%	3	0.3%	0	0.0%	32	0.1%	2	0.1%	3	0.0%	0	0.0%	0	0.0%	0	0.0%
*total*			66	6.2%	25	3.3%	43	3.8%	650	5.9%	2114	5.6%	57	2.2%	2365	8.5%	13	1.4%	10	1.8%	75	10.7%
Endo-hemicellulases																						
GH8	endo-xylanases	PF02011	1	0.1%	1	0.1%	7	0.6%	85	0.8%	174	0.5%	46	1.8%	329	1.2%	4	0.4%	1	0.2%	5	0.7%
GH10	endo-1,4-β xylanase	PF00331	15	1.4%	5	0.7%	8	0.7%	258	2.3%	2664	7.1%	25	1.0%	1025	3.7%	7	0.7%	11	2.0%	46	6.5%
GH11	xylanase	PF00457	4	0.4%	2	0.3%	4	0.4%	20	0.2%	244	0.6%	1	0.0%	165	0.6%	1	0.1%	0	0.0%	14	2.0%
GH12	endoglucanase & xyloglucan hydrolases	PF01670	3	0.3%	1	0.1%	3	0.3%	0	0.0%	0	0.0%	0	0.0%	0	0.0%	0	0.0%	0	0.0%	0	0.0%
GH26	β- mannanase & xylanase	PF02156	5	0.5%	6	0.8%	10	0.9%	103	0.9%	537	1.4%	11	0.4%	369	1.3%	5	0.5%	5	0.9%	15	2.1%
GH28	galacturonases	PF00295	11	1.0%	8	1.1%	20	1.8%	242	2.2%	244	0.6%	69	2.7%	472	1.7%	5	0.5%	2	0.4%	6	0.9%
GH53	endo-1,4-β-galactanase	PF07745	4	0.4%	1	0.1%	5	0.4%	88	0.8%	1066	2.8%	9	0.3%	0	0.0%	17	1.8%	9	1.6%	12	1.7%
*total*			43	4.1%	24	3.2%	57	5.0%	796	7.2%	4929	13.1%	161	6.2%	2360	8.5%	39	4.1%	28	5.0%	98	13.9%
Debranching enzymes																						
GH51	α-L-arabinofuranosidase	NA	14	1.3%	7	0.9%	13	1.1%	239	2.2%	0	0.0%	22	0.8%	0	0.0%	64	6.7%	12	2.2%	18	2.6%
GH54	α-L-arabinofuranosidase	PF09206	0	0.0%	3	0.4%	0	0.0%	13	0.1%	111	0.3%	0	0.0%	0	0.0%	1	0.1%	0	0.0%	0	0.0%
GH62	α-L-arabinofuranosidase	PF03664	3	0.3%	0	0.0%	0	0.0%	0	0.0%	0	0.0%	2	0.1%	1	0.0%	0	0.0%	0	0.0%	0	0.0%
GH67	α-glucuronidase	PF07477 PF07488	0	0.0%	3	0.4%	8	0.7%	0	0.0%	1090	2.9%	5	0.2%	120	0.4%	0	0.0%	5	0.9%	10	1.4%
GH78	α-L-rhamnosidase	PF05592	0	0.0%	19	2.5%	7	0.6%	413	3.7%	426	1.1%	73	2.8%	1260	4.5%	34	3.6%	25	4.5%	0	0.0%
*total*			17	1.6%	32	4.3%	28	2.5%	665	6.0%	1627	4.3%	102	3.9%	1381	5.0%	99	10.3%	42	7.5%	28	4.0%
Oligosaccharide-degrading enzymes																						
GH1	β-glucosidase & other β-linked dimers	PF00232	26	2.5%	23	3.1%	46	4.0%	103	0.9%	331	0.9%	294	11.4%	253	0.9%	10	1.0%	61	11.0%	22	3.1%
GH2	β-galactosidases & other β-linked dimers	PF02836 PF00703 PF02837	59	5.6%	22	2.9%	37	3.3%	917	8.3%	942	2.5%	66	2.5%	1436	5.2%	186	19.4%	24	4.3%	23	3.3%
GH3	mainly βglucosidases	PF00933	69	6.5%	39	5.2%	70	6.2%	804	7.3%	5448	14.5%	219	8.5%	2844	10.2%	176	18.4%	72	12.9%	69	9.8%
GH29	α-L-fucosidase	PF01120	15	1.4%	11	1.5%	9	0.8%	376	3.4%	899	2.4%	70	2.7%	939	3.4%	74	7.7%	2	0.4%	0	0.0%
GH35	β-galactosidase	PF01301	12	1.1%	7	0.9%	9	0.8%	123	1.1%	468	1.2%	32	1.2%	158	0.6%	12	1.3%	3	0.5%	3	0.4%
GH38	α-mannosidase	PF01074 PF07748	15	1.4%	12	1.6%	22	1.9%	81	0.7%	90	0.2%	18	0.7%	272	1.0%	17	1.8%	3	0.5%	11	1.6%
GH39	β-xylosidase	PF01229	7	0.7%	6	0.8%	4	0.4%	89	0.8%	159	0.4%	6	0.2%	315	1.1%	2	0.2%	1	0.2%	3	0.4%
GH42	β-galactosidase	PF02449 PF08533 PF08532	16	1.5%	14	1.9%	24	2.1%	37	0.3%	207	0.6%	54	2.1%	374	1.3%	11	1.1%	8	1.4%	24	3.4%
GH43	arabinases & xylosidases	PF04616	56	5.3%	28	3.7%	52	4.6%	894	8.1%	2313	6.2%	185	7.1%	0	0.0%	61	6.4%	10	1.8%	16	2.3%
GH52	β-xylosidase	PF03512	0	0.0%	0	0.0%	2	0.2%	0	0.0%	0	0.0%	0	0.0%	0	0.0%	0	0.0%	0	0.0%	3	0.4%
*total*			275	26.0%	162	21.6%	275	24.2%	3424	31.0%	10857	28.9%	944	36.4%	6591	23.7%	549	57.4%	184	33.0%	174	24.7%
*total plant plysaccharides targeting GHs*			401	37.9%	243	32.4%	403	35.5%	5535	50.1%	19527	52.0%	1264	48.8%	12697	45.7%	700	73.1%	264	47.4%	375	53.3%
*total GHs*			1059		750		1136		11038		37563		2590		27755		957		557		704	

GHs are grouped according to the major functional roles as classified in Allgaier *et al*.^62^. For each GH family, the total number and the percentage on total GHs detected in the respective sample were shown.

^a^Searches for glycoside hydrolases were performed by using HMMER hmmsearch with Pfam_Is HMMs (full-length models) to identify complete matches to the family, which were named in accordance with the CAZy nomenclature scheme.

^b^The detection and assignment of glycoside hydrolases were performed by BLAST-based procedures against the CAZy database.
